# Tumor-infiltrating CCR2^+^ inflammatory monocytes counteract specific immunotherapy

**DOI:** 10.3389/fimmu.2023.1267866

**Published:** 2023-10-02

**Authors:** Joschka Bartneck, Ann-Kathrin Hartmann, Lara Stein, Danielle Arnold-Schild, Matthias Klein, Michael Stassen, Federico Marini, Jonas Pielenhofer, Sophie Luise Meiser, Peter Langguth, Matthias Mack, Sabine Muth, Hans-Christian Probst, Hansjörg Schild, Markus Philipp Radsak

**Affiliations:** ^1^ III^rd^ Department of Medicine - Hematology, Oncology, University Medical Center of the Johannes Gutenberg-University, Mainz, Germany; ^2^ Institute of Immunology, University Medical Center of the Johannes Gutenberg-University, Mainz, Germany; ^3^ Institute of Medical Biostatistics, Epidemiology and Informatics (IMBEI), University Medical Center of the Johannes Gutenberg-University, Mainz, Germany; ^4^ Institute of Pharmaceutical and Biomedical Sciences of the Johannes Gutenberg-University, Biopharmaceutics and Pharmaceutical Technology, Mainz, Germany; ^5^ University Hospital Regensburg, Department Nephrology, Regensburg, Germany

**Keywords:** cancer immunotherapy, transcutaneous immunization, tumor micro environment (TME), immune evasion, CCR2 monocytes +

## Abstract

Tumor development and progression is shaped by the tumor microenvironment (TME), a heterogeneous assembly of infiltrating and resident host cells, their secreted mediators and intercellular matrix. In this context, tumors are infiltrated by various immune cells with either pro-tumoral or anti-tumoral functions. Recently, we published our non-invasive immunization platform DIVA suitable as a therapeutic vaccination method, further optimized by repeated application (DIVA^2^). In our present work, we revealed the therapeutic effect of DIVA^2^ in an MC38 tumor model and specifically focused on the mechanisms induced in the TME after immunization. DIVA^2^ resulted in transient tumor control followed by an immune evasion phase within three weeks after the initial tumor inoculation. High-dimensional flow cytometry analysis and single-cell mRNA-sequencing of tumor-infiltrating leukocytes revealed cytotoxic CD8^+^ T cells as key players in the immune control phase. In the immune evasion phase, inflammatory CCR2^+^ PDL-1^+^ monocytes with immunosuppressive properties were recruited into the tumor leading to suppression of DIVA^2^-induced tumor-reactive T cells. Depletion of CCR2^+^ cells with specific antibodies resulted in prolonged survival revealing CCR2^+^ monocytes as important for tumor immune escape in the TME. In summary, the present work provides a platform for generating a strong antigen-specific primary and memory T cell immune response using the optimized transcutaneous immunization method DIVA^2^. This enables protection against tumors by therapeutic immune control of solid tumors and highlights the immunosuppressive influence of tumor infiltrating CCR2^+^ monocytes that need to be inactivated in addition for successful cancer immunotherapy.

## 1 Introduction

Despite major treatment advances in cancers by various approaches including polychemotherapy, surgery, radiotherapy or combinations thereof resulting in improved tumor control, survival or even cure, the treatment of cancer remains a major health burden due primary or secondary development of therapy resistance. In this context, the tumor microenvironment (TME) has a key role in the regulation of the susceptibility of cancer cells to therapeutics, especially with respect to immunotherapies. The TME includes beyond tumor cells numerous other cell types, such as fibroblasts, endothelial cells, and various immune cells, as well as secreted mediators in addition to blood vessels and structure-giving extracellular matrix (1).

Beyond the suppression of immune inhibitory signals by immune checkpoint inhibition *via* PD-1/PD-L1 or CTLA4, the use of cancer vaccines that induce the generation of high-quality tumor-specific T cells is a promising tool to mount immune responses against tumor specific target antigens, a field of intense investigation (2). Here therapeutic approaches are needed that specifically sensitize the host immune system to the tumor, able to specifically address the targets in the complex immune-inhibitory network of the TME. This comprises a major challenge for the immune mediated elimination of cancer cells due to its heterogeneity and multitude of immunosuppressive factors (3). Therefore, the characterization of immunosuppressive mechanisms within the TME is of central importance in the development of immunotherapeutic vaccination approaches. In this regard, non-invasive immunization strategies applying a vaccine onto the intact skin (transcutaneous immunization; TCI) are of increasing interest. In comparison to conventional vaccines, TCI targets skin-resident professional antigen-presenting cells (APC), inducing efficient T cell priming in draining lymph nodes and mounting potent anti-tumor T cell responses. Since the primary description of TCI using cholera toxin by Glenn et al. in 1998 (4), various approaches have been developed to deliver antigens and adjuvants over the skin barrier, distinguishing between active and passive approaches (5). In our approach, we use the passive transport of antigenic peptides together with the Toll-like receptor 7 (TLR7) agonist imiquimod (IMQ) (6) and the anti-psoriatic agent dithranol (also known as anthralin) onto the intact skin (DIVA, dithranol imiquimod-based vaccination). DIVA initiates superior primary CTL responses and a long-lasting memory T cell response after a single treatment (7). Further optimizing this vaccination protocol towards a more effective boost strategy, termed DIVA^2^, generates potent primary and memory immune responses, crucial for immunotherapeutic vaccination against cancer (8).

In our present work, we report the influence of therapeutic vaccination on the TME by DIVA^2^ which provides transient tumor immune control. As major counter regulator of immunological tumor control, we identify tumor-infiltrating immunosuppressive monocytes contributing to immune evasion in this setting. Upon treatment with DIVA^2^ as therapeutic cancer vaccine, transitional immune control of tumor growth was achieved by the induction of OVA_257-264_ -specific highly functional CD8^+^ T cells, characterized by IFN-γ production and cytotoxic gene signature. However, this was followed by secondary failure and tumor outgrowth. Flow cytometry and scRNA-seq analysis revealed CCR2^+^ monocytes to be only detectable during immune evasion, but not during immune control. When the monocyte-depleting anti-CCR2 antibody MC-21 was injected after DIVA^2^, a temporary reduction in tumor growth was observed, suggesting that the immunosuppressive phenotype of the CCR2^+^ tumor-infiltrating monocytes is responsible for the failure of tumor specific T cells to eradicate tumors. In summary, we present a characterization of the TME upon cancer immunotherapy by therapeutic vaccination through transcutaneous immunization. Specifically, we highlight CCR2^+^ monocytes as key players in the TME that potentially serve as new targets for optimized immunotherapy using DIVA.

## 2 Results

### 2.1 DIVA^2^ induces transient tumor immune control that turns into immune evasion

**Figure 1:**
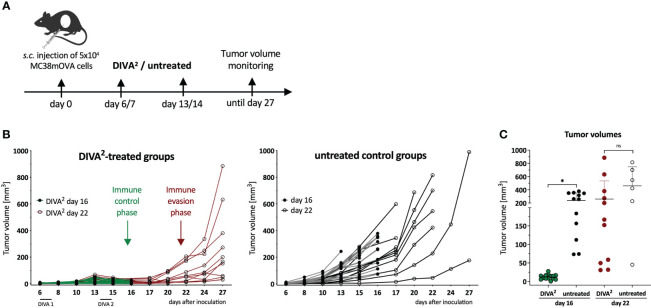
Therapeutic DIVA^2^ induces transient tumor control that turns into immune evasion. **(A)** Application pattern for DIVA^2^ in a therapeutic tumor setting. Mice were immunized twice one and two weeks after tumor implantation using DIVA^2^ or left untreated and the tumor volume was monitored three times per week. In this setting, two independent experiments were performed. **(B)** Tumor volumes were assessed three times per week until day 16 (green lines) or until day 27 (red lines). Every curve represents the tumor volume of one individual animal (n=11-15). **(C)** Tumor volumes during immune control phase on day 16 and immune evasion phase on day 22 were displayed. Visualized are individual data points, mean and SD. *p < 0.05 by two-way ANOVA with Sidak’s multiple comparisons test and one-way ANOVA with Kruskal-Wallis test, when sample numbers were different.

Our transcutaneous immunization approach DIVA^2^ is a non-invasive immunization technique generating highly specific anti-tumor T cell responses (8). In this study, we wanted to characterize the composition of the TME upon therapeutic vaccination by DIVA^2^. The colorectal tumor model MC38 is an established tumor model for optimizing immunotherapeutic approaches (9). MC38 is a so called “hot” tumor, characterized by rich immune cell infiltration and susceptible to immunotherapy. To reveal the biological relevance of DIVA^2^ in a therapeutic tumor setting, we transfected MC38 cells with ovalbumin (MC38mOVA). Therefore, we injected C57BL/6 mice with ovalbumin-expressing MC38 (MC38mOVA) tumor cells and applied DIVA^2^ with ovalbumin peptides (OVA_257-264_ and OVA_323-337_) when tumors were palpable (**Figure 1A**). Compared to untreated mice, DIVA^2^-treatment reduced the tumor volume, resulting in immune control that was maintained for over two weeks. However, this phase of immune control quickly turned into immune evasion, reflected by strongly increasing tumor volumes (**Figure 1B**). During immune control, the tumor volumes were significantly reduced compared to untreated mice, but this difference was rapidly lost resulting in no significant differences in tumor size over the next 6 days (**Figure 1C**). As there is a variety of molecular mechanisms of tumor cells to escape immune control, we interrogated the most common ways of immune evasion and asked for the loss of antigen presented to T cells *via* MHC class I molecules on the surface of tumor cells. This occurs by downregulating proteins involved in the antigen processing or presentation machinery, resulting in a decrease or loss of presented antigen. Therefore, we investigated whether there is a decrease or loss of the OVA_257-264_ epitope on the surface of MC38mOVA cells during DIVA^2^-induced immune control that could cause immune evasion. We performed a proliferation assay of OT-I transgenic T cells recognizing the OVA_257-264_ epitope in the context of H2-K^b^ on *ex vivo* MC38mOVA tumor cells (**Supplementary Figure 1A**). DIVA^2^ induced up to 40% OVA_257-264_-specific tumor-infiltrating T cells with highly activated phenotype (**Supplementary Figures 1B, C**). However, OT-I T cells proliferated after co-culture with *ex vivo* MC38mOVA cells, regardless of the timepoint of tumor cell isolation and whether mice were immunized (**Supplementary Figure 1D**) suggesting that antigen loss as a possible reason for immune evasion after initial DIVA^2^-induced immune control can be excluded as well as the lack of access of specific CTLs to the TME.

### 2.2 DIVA^2^-induced immune control is accompanied with infiltration of OVA-specific T cells and absence of inflammatory monocytes

**Figure 2:**
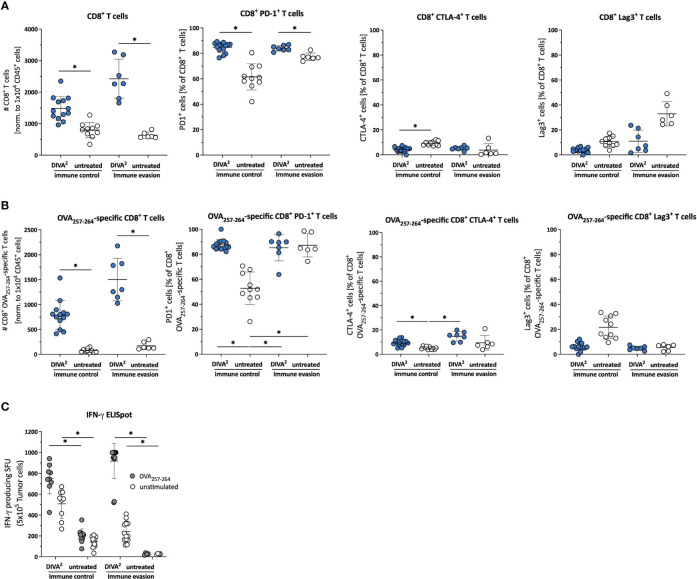
DIVA^2^-induced tumor-infiltrating CD8^+^ T cells exhibit an activated and functional phenotype. **(A)** Cell counts of tumor-infiltrating CD45^+^ cells, CD8^+^ T cells, **(B)** specific CD8^+^ T cells and frequencies of their PD-1, CLTA-4 and Lag-3 expression were assessed by flow cytometry during immune control (day 16) and immune evasion (day 22) (n=11-15). Visualized are individual data points, mean and SD. **(C)** *Ex vivo* tumor cell suspensions were restimulated for 20 h with OVA_257-264_ or left unstimulated to determine IFN-γ production by ELISpot assay. p< 0.05 by one-way ANOVA with Kruskal-Wallis test. The Flow cytometric gating strategy is shown in **Supplementary Figure 3**.

**Figure 3:**
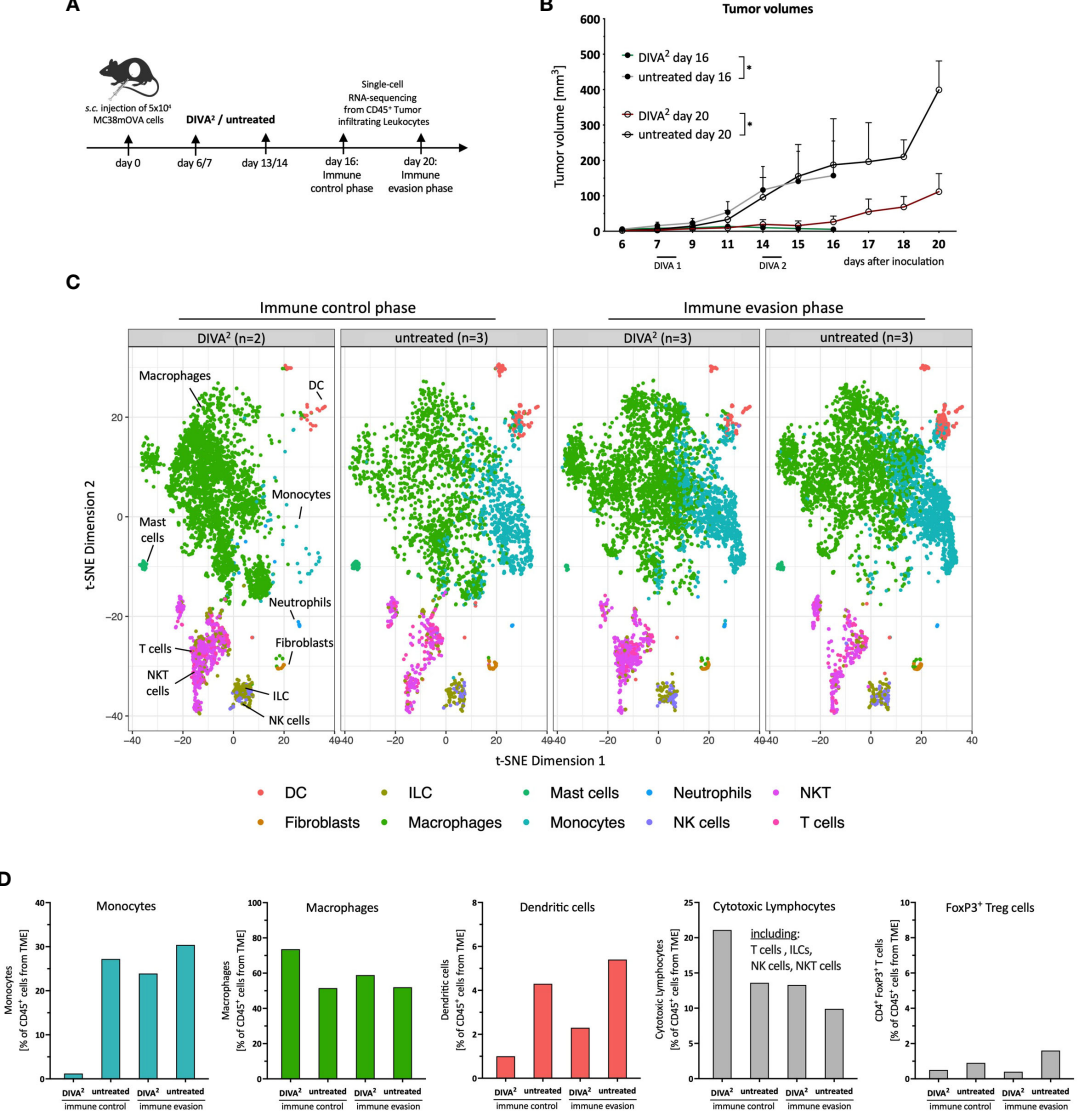
Single-cell RNA-sequencing analysis reveals monocytes to be absent during DIVA^2^-induced immune control. **(A)** Application pattern for DIVA^2^ in a therapeutic tumor setting. Tumor cell suspensions were prepared during immune control (day 16) or immune evasion (day 20). Tumor-infiltrating leukocytes were prepared by MACS isolation of CD45^+^ cells. **(B)** Tumor volumes during immune control (day 16, green line) and immune evasion (day 20, red line). Visualized are the means and SD. *p<0.05 by two-way ANOVA with Sidak’s multiple comparisons test. Statistics were analyzed on day 16 and day 20, compared to the non-immunized control groups. **(C)** scRNA-seq-based t-SNE plots of tumor-infiltrating leukocytes, merged per condition (n=2-3). Cell types were predicted based on the immgen database annotation *immgen main*. **(D)** Quantitative distribution of tumor-infiltrating leukocytes per immune cell type and condition.

To gain more detailed information on the TME, we examined the CD45^+^ tumor-infiltrating leukocytes by high dimensional flow cytometry during immune control (day 16) and immune evasion (day 22/27). In the immune control phase, DIVA^2^ induced significant higher numbers of CD8^+^ T cells and especially OVA_257-264_-specific CD8^+^ T cells, characterized by a high expression of PD1 and a very low expression of CTLA-4 and Lag3, suggesting a highly activated, but not exhausted state (**Figures 2A, B**). Furthermore, we detected the functional phenotype of OVA_257-264_-specific CD8^+^ T cells by specific restimulation of whole tumor cell suspensions in an IFN-γ ELISpot assay (**Figure 2C**). However, since tumor volumes still increased after initial immune control, there must be immunosuppressive factors in the TME hindering the cytotoxic lymphocytes from eliminating tumor cells completely. To find out more precisely which mechanisms in the TME prevent a successful immunotherapy by DIVA^2^, we set out to perform single-cell RNA-sequencing (scRNA-seq) of the tumor-infiltrating leukocytes during immune control and evasion (**Figure 3A, B**). We assigned single cells to immune cell types based on the immgen database annotation *immgen main* and visualized them in t-distributed stochastic neighbor Embedding algorithm (t-SNE) plots.

Tumor-infiltrating leukocytes isolated during immune evasion from DIVA^2^-treated and untreated mice clustered very similarly. In contrast, tumor-infiltrating leukocytes isolated during immune control clustered differently across conditions (**Figure 3C**). Strikingly, the t-SNE plot showed that during immune control, a monocyte population of the DIVA^2^-treated group was merely absent which was accompanied by a higher proportion of macrophages (**Figure 3C**). In contrast, monocytes made up about 30% of the tumor-infiltrating leukocytes in the untreated group. However, in the immune evasion phase we detected this monocyte population in both conditions. Furthermore, we detected fewer DCs in the DIVA^2^-treated group. Since regulatory T cells (Treg) can act immunosuppressive on cytotoxic lymphocytes in the TME, we analyzed the proportions of FoxP3^+^ Tregs. However, we did not detect any significant differences in any of the respective conditions. As expected, DIVA^2^ induced a significant increase in cytotoxic lymphocytes (CLs) in the immune control phase, including T cells, NK cells, NKT cells and ILCs. However, the number of cytotoxic lymphocytes decreased significantly until immune evasion, suggesting a decreasing cytotoxic capacity and thus anti-tumor effect (**Figure 3D**). The decreased frequency of CLs in the immune evasion phase accompanied by the increased abundance of monocytes in the DIVA^2^-treated group suggesting an immunosuppressive effect on CLs, a property associated with inflammatory CCR2^+^ monocytes [reviewed by (10)].

### 2.3 DIVA^2^-induced CD8^+^ T cells mainly mediate cytotoxicity but also show slight exhaustion

**Figure 4:**
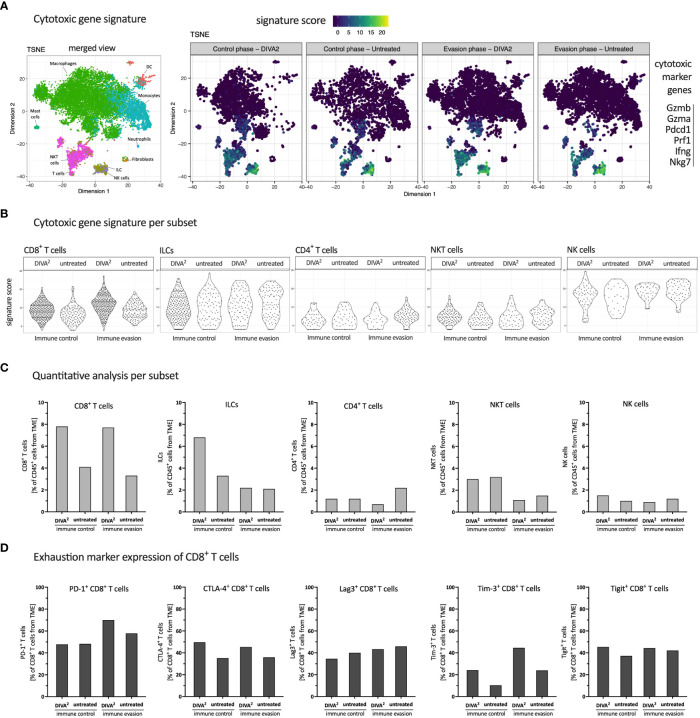
DIVA^2^ treatment induced mainly cytotoxic CD8^+^ T cells with a mild exhaustion characteristic. **(A)** scRNA-seq-based t-SNE plots of tumor-infiltrating leukocytes showing signature score of cytotoxic gene signature. **(B)** Signature score of cytotoxic gene signature, split by cytotoxic lymphocyte subtype. **(C)** Quantitative distribution of cytotoxic lymphocyte subtypes. **(D)** scRNA-seq-based expression analysis of indicated exhaustion marker genes by CD8^+^ T cells. All samples are merged per condition (n=2-3).

To characterize the phenotype of CLs in more detail, we analyzed the various subsets for the expression of cytotoxic gene signatures based on the scRNA-seq data. The t-SNE plots showed that the expression of cytotoxic marker genes is essentially restricted to T cells, NKT cells, NK cells and ILCs in relation to all tumor-infiltrating leukocyte populations. We observed that DIVA^2^ induced a larger population of T cells expressing the cytotoxic gene signature compared to untreated during both immune control and immune evasion (**Figure 4A**). To highlight the differences in lymphocytes expressing the cytotoxic gene signature, we analyzed the expression intensities and proportions relative to tumor-infiltrating leukocytes for each cytotoxic lymphocyte subtype separately (**Figures 4B, C**). The expression analysis showed that the DIVA^2^-induced CD8^+^ T cells also largely expressed cytotoxic marker genes. This phenotypic pattern increased from immune control to immune evasion. In general, these findings confirm the results of the flow cytometry-based TME analysis and the IFN-γ ELISpot of the tumor cell suspensions (**Figure 2**). The ILCs, detected in greater numbers during immune control, also expressed cytotoxic marker genes to a large extent, suggesting an ILC type 1 phenotype possibly contributing to immune control (11). However, this effect was limited in time, as their number decreased until immune evasion. For CD4^+^ T cells, the signature score was similar in both conditions and time points, but the number of cells had increased in the untreated mice until immune evasion. Moreover, the average signature score was lower compared to other subtypes suggesting a lower cytotoxic activity of CD4^+^ T cells. In addition, NKT cells were detected in equal frequencies regardless of treatment during immune control. However, upon DIVA^2^ treatment, NKT cells showed a slightly increased signature score, indicating a more pronounced cytotoxic phenotype. The number of NKT cells was decreased by half during immune evasion in both conditions suggesting they also could eliminate less tumor cells from the onset of immune evasion. NK cells were represented in the least cell number of cytotoxic lymphocytes. However, NK cells had the highest averaged signature score in the expression analysis, indicating their contribution in eliminating tumor cells. Taken together, these observations show that CD8^+^ T cells and ILCs highlighted in the quantitative analysis were also strongly expressing cytotoxic marker genes, while NKT cells, NK cells and CD4^+^ T cells were less likely to contribute to cytotoxicity. The decrease in the total cell number of cytotoxic lymphocytes until immune evasion might indicate tumor progression and thus the switch from immune control to immune evasion.

Although the expression of cytotoxic marker genes is a prerequisite for the elimination of tumor cells, T cells can exhibit the state of exhaustion. Therefore, we analyzed CD8^+^ T cells for the expression of exhaustion markers which indicates suppressed effector T cell functions. We performed a gene expression analysis with the exhaustion marker genes PD-1, CTLA-4, Lag3, Tim-3 and Tigit (**Figure 4D**) (12). PD-1 and Tim-3 expression increased from immune control to immune evasion in the DIVA^2^-treated group, suggesting a continued antigen contact and activation state of these T cells. CTLA-4, Lag3 and Tigit expression after DIVA^2^ were comparable at both timepoints. The expression of exhaustion marker genes suggests reduced effector T cell functions. In addition to the decreasing total number of CLs at the time of immune evasion compared to immune control (**Figure 3D**), exhaustion of CD8^+^ T cells potentially represents a second mechanism for increased tumor growth after initial tumor immune control.

### 2.4 CCR2^+^ Monocytes infiltrating the TME during immune evasion express immunosuppressive marker genes

**Figure 5:**
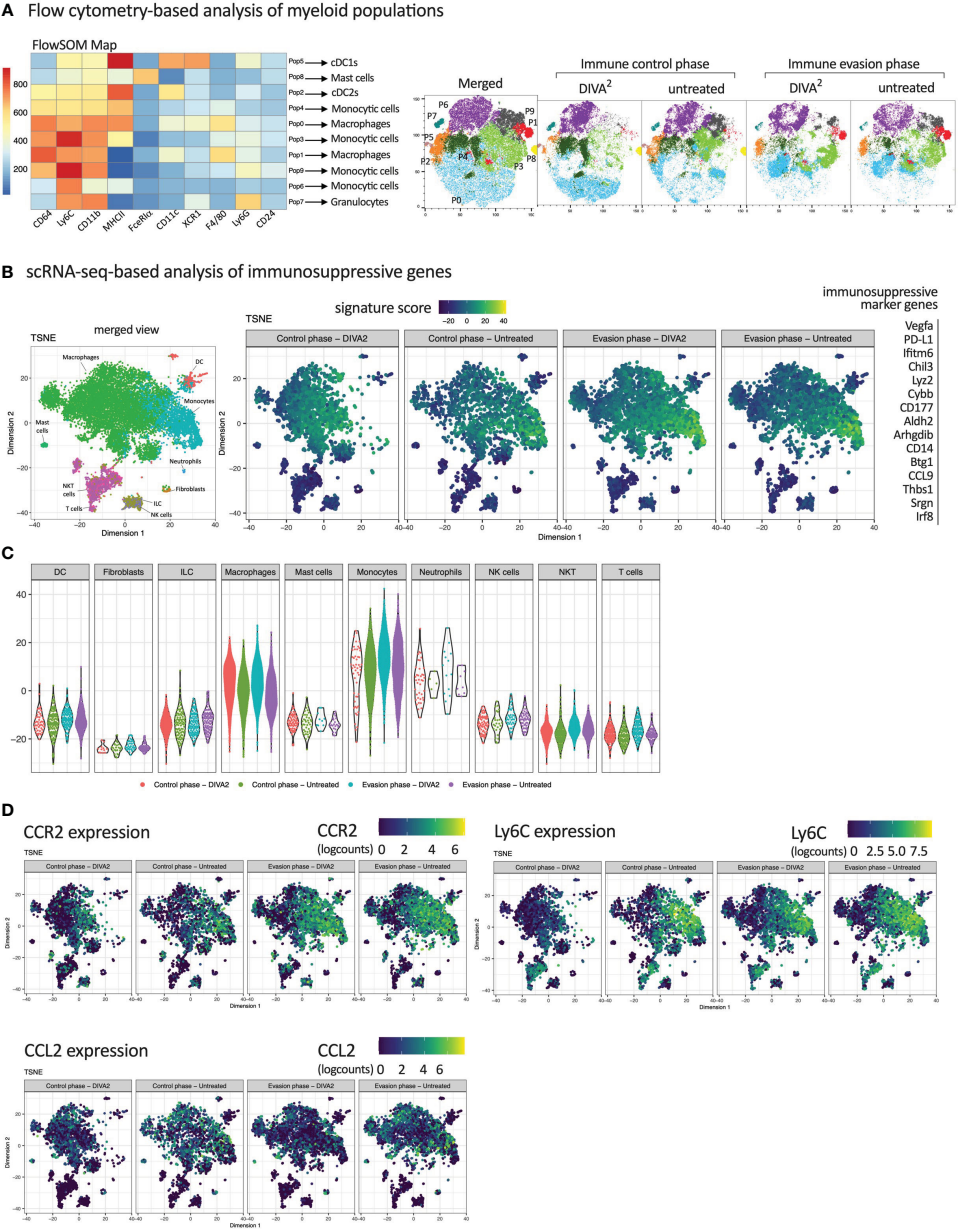
Inflammatory CCR2^+^ Monocytes infiltrating the TME during immune evasion express immunosuppressive marker genes. **(A)** FlowSOM Map of CD45^+^ tumor-infiltrating immune cells and their predicted cell types. Cells were pre-gated on living cells, single cells, Lineage^-^ cells and CD45^+^ cells. Expression intensities were relatively set by the FlowSOM algorithm. t-SNE plots of CD45^+^ tumor-infiltrating immune cells, merged per condition (n=11-15). FACS Markers included in the t-SNE calculation are analogous to the markers in the FlowSOM map. For coloring, FlowSOM populations were applied onto the t-SNE plots. **(B)** scRNA-seq-based t-SNE plots of tumor-infiltrating leukocytes showing signature score of immunosuppressive gene signature. **(C)** Signature score of immunosuppressive gene signature, split by immune cell types. **(D)** scRNA-seq-based t-SNE plots of tumor-infiltrating leukocytes showing expression of indicated genes. All scRNA-seq samples are merged per condition (n=2-3). Flow cytometric gating strategies are shown in **Supplementary Figure 3**. The flow cytometric gating strategy until gating of CD45^+^ lineage^-^ cells was performed according to the gating strategy of **Figure 2**.

To analyze the impact of the myeloid compartment within the TME on the immunosuppression of T cells, we calculated and visualized myeloid populations based on high dimensional flow cytometry data using FlowSOM and t-SNE algorithms. Based on the fluorescence intensity of the myeloid flow cytometry markers in the FlowSOM heatmap, we assigned the predicted populations to their respective cell types (**Figure 5A**). Strikingly, we found four different monocyte populations (P3, P4, P6 and P9) and two different macrophage populations (P0 and P1), in various differentiation stages, indicated by their MHCII and Ly6C expression intensities. While the t-SNE clustering during immune evasion is very similar between DIVA^2^ and untreated, the corresponding t-SNE plots during immune control differ remarkably. These differences mainly relate to the monocytic populations P3 and P9 and the macrophage population P0. These data suggest that the composition of the myeloid compartment is altered when mice are treated by DIVA^2^. Due to the limited number of markers in the flow cytometry analysis, we were not able to determine a functional phenotype of the monocytes. Therefore, we analyzed the expression of immunosuppressive marker genes based on the *ex vivo* scRNA-seq data from tumor tissue (**Figure 5B**). The strongest expression of these marker genes was observed in the monocyte population which was almost absent during immune control phase. To further examine the extent to which the expression of the immunosuppressive marker genes relates to monocytes, we plotted the expression intensity for each cell type (**Figure 5C**). As already indicated in the t-SNE plots, macrophages also expressed the immunosuppressive marker genes, but at a lower signature score than monocytes. Only neutrophil granulocytes had a signature score comparable to monocytes but were represented in a very small cell number. These results indicate that monocytes infiltrating the TME after the immune control phase exhibit an immunosuppressive phenotype. Hence, they can contribute significantly to immunosuppression of pro-inflammatory immune cells within the TME, for example by causing exhaustion of CD8^+^ T cells. To verify the immunosuppressive effect of these monocytes in an *in vivo* experiment, we examined the monocytes for a potential target suitable for depletion in a tumor setting. The CCL2/CCR2 axis plays a crucial role in the recruitment of monocytic cells to the tumor site. The chemokine CCL2 can be expressed in the TME by stroma cells, endothelial cells, tumor cells or leukocytes (13), forming a CCL2 gradient within the tissue. Cells expressing the CCL2-receptor CCR2 on their cell surface can migrate along a CCL2 gradient to the peripheral tumor site. Once in the TME, these cells can contribute to the suppression of pro-inflammatory cells. The t-SNE plot split by conditions showed that besides macrophages, mainly monocytes expressed CCR2 (**Figure 5D**). CCL2-expression was stronger in the untreated group during immune control and immune evasion. Since monocytes were almost absent in the DIVA^2^-treated group during immune control, the total number of CCL2-expressing cells was thus also lower. However, we detected a strong increase in CCR2^+^ monocytes in the immune invasion phase, regardless of treatment, suggesting that these monocytes migrate into the TME *via* CCL2/CCR2 signaling. Notably, these monocytes expressed Ly6C, confirming the classification as inflammatory monocytes capable of mediating immunosuppression (**Figure 5D**). Taken together, we identified inflammatory monocytes as key players in the immunosuppressive mechanisms most likely influencing T cell functions in the TME.

### 2.5 Depletion of CCR2^+^ Monocytes in a therapeutic tumor setting leads to decreased tumor growth demonstrating their immunosuppressive capacity

**Figure 6:**
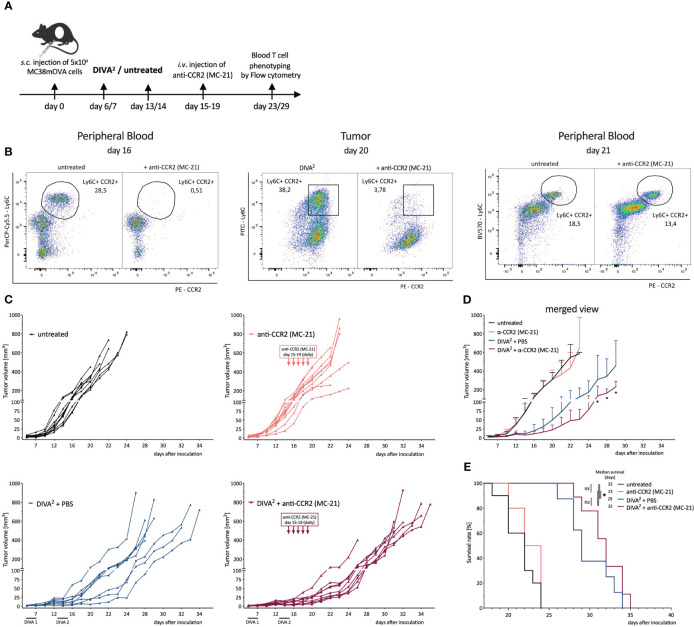
Depletion of CCR2^+^ Monocytes in a therapeutic tumor setting leads to a decreased tumor growth demonstrating their immunosuppressive capacity. **(A)** Schematic overview of the application pattern for Boost DIVA in a therapeutic tumor setting. DIVA^2^-treated or untreated mice were *i.v.* injected with anti-CCR2 antibody MC-21 from day 15-19 (20 µg daily) or left untreated (n=4-9). **(B)** Representative flow cytometry dot plots of LY6C^+^ CCR2^+^ peripheral blood cells of an untreated and anti-CCR2 treated mouse. **(C)** Tumor volumes were assessed three times per week. Every curve represents the tumor volume of one individual mouse. **(D)** Tumor volumes visualized as mean and SD per condition. **(E)** Kaplan-Meier survival curve. p < 0.05 by two-way ANOVA with Sidak’s multiple comparisons test and one-way ANOVA with Kruskal-Walli’s test, when sample numbers were different. Comparisons of survival curves were performed by Log-rank (Mantel-Cox) test.

Next, we characterized the anti-inflammatory phenotype of CCR2^+^ Tumor-infiltrating monocytes, absent in DIVA^2^-treated mice during immune control, but detectable during immune evasion. To evaluate their tumor promoting capacity we depleted CCR2^+^ cells in a therapeutic tumor setting with the anti-CCR2 antibody MC-21 and hypothesized a decrease of tumor growth after depletion. As we only detected the monocytes after the immune control phase, we started the MC-21 treatment on day 15 (**Figure 6A**). We verified the depletion of Ly6C^high^ CCR2^+^ peripheral blood monocytes 24 h after the first injection. As expected, we observed a depletion of Ly6C^high^ CCR2^+^ peripheral blood monocytes. This depletion was accompanied by an almost complete depletion of CCR2^+^ monocytes in the tumors on day 20. These findings suggest that CCR2^+^ monocytes infiltrate the TME from peripheral blood, but that infiltration can be prevented by the anti-CCR2 antibody MC-21. However, we detected CCR2^+^ monocytes in the peripheral blood again 48 h after the last MC-21 injection at day 21, administered on 5 consecutive days (**Figure 6B**). Combining DIVA^2^ and MC-21 treatment in a therapeutic tumor setting reduced the tumor growth significantly, compared to DIVA^2^ alone. However, this effect was limited and lasted only until about 5 days after the last MC-21 injection. Thereafter, we observed that the tumor volume increased more rapidly. Since the monocytes were detectable in the blood about 24 h after the last injection, these findings suggest that the CCR2^+^ monocytes exhibit a tumor-promoting effect, which unfolds again when the depletion effect runs out. Notably, treatment with MC-21 alone did not induce a reduction in tumor growth, indicating that depletion of tumor-promoting monocytes no longer has an effect when started in the later evasion phase (**Figures 6C, D**). In this context, the effect of MC-21 alone on the tumor growth at an earlier stage cannot be predicted. The combined immunotherapy prolonged the median survival of the mice to 32 days, confirming the enhanced anti-tumoral effect (**Figure 6E**). However, the combined immunotherapy did not significantly enhance the overall survival compared to DIVA^2^-treatment alone. We further clarified if this anti-tumoral effect was due to depletion of tumor promoting CCR2^+^ monocytes or rather to an altered T cell immune response. For this purpose, we functionally characterized the circulating T cells at different time points. Surprisingly, despite decreased tumor growth in MC-21-treated animals, we found even fewer CD8^+^ T cells and Ova_257-264_-specific CD8^+^ T cells. These cells produced similar amounts of IFN-γ, TNF-α and KLRG-1, suggesting a functional, non-senescent phenotype. Even though CD8^+^ T cells can express CCR2 (14), depletion of CCR2^+^ cells did not induce depletion of T cells, as we found no difference in T cell count after treatment with MC-21 alone compared to untreated mice (**Supplementary Figure 2A**). Addressing CD4^+^ T cells, we also observed no differences between DIVA^2^-treated and untreated mice (**Supplementary Figure 2B**). Taken together, the findings highlight the role of CCR2^+^ tumor-infiltrating monocytes in contributing to a tumor-promoting microenvironment. The associated tumor growth could only be slowed down temporarily by the anti-CCR2 antibody MC-21, demonstrating the need for alternative substances to permanently deplete tumor-promoting monocytes.

## 3 Discussion

Therapeutic vaccines aim to induce tumor regression and long-lasting tumor control (15) by inducing highly specific T cell-mediated immune responses to tumor antigens (16). Recently, we established a novel transcutaneous immunization approach DIVA, based on imiquimod and dithranol (7), capable to induce tumor rejection after further optimization (DIVA^2^) (8). However, in a therapeutic setting DIVA^2^ merely mediates transient protection during an immune control phase followed by a tumor evasion phase with tumor outgrowth (**Figure 1B**), indicating that our potent vaccination approach alone merely is insufficient for tumor rejection.

To understand the underlying mechanisms driving tumor progression, we compare the TME during the phase of tumor control and tumor progression. Firstly, we can exclude the loss of antigen on MC38mOVA tumor cells as a potential reason for immune evasion, using an *in vitro* proliferation assay with transgenic T cells (OT-1 T cells; **Supplementary Figure 1D**) (17, 18), as no impact on the proliferation of OT-1 T cells after co-cultivation with *ex vivo* MC38mOVA tumor cells was detectable. Secondly, to understand the mechanisms driving immune control after DIVA^2^, we confirm the tumor (OVA_257-264_) specificity and functional phenotype of the induced CD8^+^ T cells by a pronounced cytotoxic gene expression profile and strong IFN-γ release (**Figures 2**, **4**) (19–21). IFN-γ is a key effector molecule for the cytotoxic function of CD8^+^ T cells (22) inhibiting tumor proliferation by promoting the expression of cell cycle inhibitors (p27Kip, p16 or p21) (23–25). Furthermore, IFN-γ induces apoptosis and necrosis (26) and acts as an inhibitor of angiogenesis in tumor tissue (27–29). However, in contrast to its anti-tumoral functions, IFN-γ may also exert pro-tumoral functions (30–36) by activating immune checkpoint genes such as PD-L1 or PD-L2 on tumor cells. These ligands bind to PD-1 on T cells or NK cells leading to immunosuppression (37–40). Along these lines, the strong IFN-γ production induced by DIVA^2^ may trigger pro-tumoral properties, in turn inhibiting the induced T cells. This notion is supported by our single-cell RNA-sequencing data in the immune evasion phase revealing a decrease in the population of cytotoxic lymphocytes (**Figure 3D**) mainly formed by CD8^+^ T cells, ILCs and NKT cells (**Figure 4C**). Analysis of the exhaustion markers of CD8^+^ T cells in the immune phase as well as in the immune evasion phase, show an increased expression of PD-1, Lag3 and Tim-3 in this phase, indicative of a moderate exhausted phenotype [reviewed by Catakovic et al. (41)] (**Figure 4D**). In an exhausted state, T cells are inhibited in their effector function and therefore cannot promote anti-tumor immunity, leading to tumor growth (42–45). ILCs are most abundant in the immune control phase after DIVA^2^ treatment. Clustering of this population in the t-SNE plots closely to NK cells (**Figures 3C**, **4A**) suggests an ILC1 phenotype by the cytotoxic gene marker analysis (**Figure 4B**). Collectively, DIVA^2^-induced immune control is mainly mediated by ILCs and CD8^+^ T cells. However, exaggerated IFN-γ production in this setting may promote a pro-tumoral milieu driving tumor progression. Therefore, IFN-γ cannot be regarded as a master regulator of tumor immunity and may act as a double-edged sword depending on the cellular context in the TME.

Given the persistence of the DIVA^2^-induced cytotoxic CD8^+^ T cells in the tumor and the concurrent loss of immune control, immunosuppressive mechanisms in TME must prevent tumor cell elimination and immune evasion. High dimensional flow cytometry of tumor infiltrating CD45^+^ leukocytes draws our attention to the myeloid compartment of the TME to be mainly composed of several monocyte- and macrophage populations. More detailed t-SNE analysis at individual time points after treatment revealed that the myeloid compartment during the immune control differed greatly compared to the untreated group. Interestingly, IFN-γ is also associated with the polarization of macrophages into inflammatory M1 macrophages (46). Along this line, we observe an increased infiltration of macrophages in the tumor in the immune control phase (**Figures 3D**, **5A**). In contrast, in immune evasion phase the clustering was very similar across the conditions (**Figure 5A**), suggesting a relevant impact of the myeloid compartment in the initiation of immune evasion. ScRNA-seq data of tumor-infiltrating CD45^+^ leukocytes confirmed the flow cytometry data. Notably, monocytes were nearly absent in the DIVA^2^-treated group in immune control phase compared to untreated animals. In contrast, during the immune evasion phase, monocytes are abundant regardless of treatment (**Figure 3C**). Expression analysis of immunosuppressive marker genes, associated with MDSC phenotypes (47), indicates an immunosuppressive phenotype of the monocytes infiltrating the TME in the immune evasion phase (**Figures 5B, C**).

Chemokines produced by tumor cells can drive the infiltration of immune cells into the TME and chemokine (C-C motif) ligand 2 (CCL2), also known as monocyte chemoattractant protein-1 (MCP-1) plays an important role in this context (48). CCR2 expressing monocytes are recruited along the CCL2 gradient to the peripheral tumor site (49, 50). In the TME, monocytes can further mature and develop pro-tumoral functions (51, 52) by maturing into tumor-associated macrophages (TAMs) promoting tumor growth (51, 53, 54). Pre-clinical models targeting the CCR2/CCL2 axis have already revealed an impact on tumor growth by blockade of CCR2/CCL2 binding (55). As shown in **Figure 5D**, tumor-infiltrating leukocytes display a high CCR2 as well as Ly6C expression on monocytes during immune evasion independent of treatment indicating a monocyte derived-MDSC (M-MDSC) phenotype (56). In addition, to some extent CCL2 expression was also observed in the scRNA-seq data (**Figure 5D**). The abundance of CCR2^+^ monocytes in the TME is associated with the suppression of T cells in various cancer models (57–60) shaping tumor progression because of immunosuppressive mechanisms initiated upon recruitment. In line with this, the observed CCR2^+^ monocytes expressed Irf8 (**Supplementary Figure 4**), which is associated with the induction of T cell exhaustion, further promoting tumor growth (61). Furthermore, tumor-infiltrating monocytes are known to induce the recruitment of tumor promoting Treg cells (60, 62). Nevertheless, Treg cell frequencies were comparable in all groups (**Figure 3D**) at the investigated time points suggesting no prominent impact of Tregs on the immunosuppressive mechanisms shaping tumor progression. A limitation of our studies is certainly the circumstance that we did not investigate the functional interaction of tumor-infiltrating monocytes with T cell populations. Hence, we are at present unable conclude whether these cells are truly responsible for tumor progression in the immune evasion phase. Further studies are needed to confirm this and pinpoint the underlying mechanisms to disclose the full potential of specific cancer immunotherapies.

To gain insight on the biological relevance of CCR2^+^ monocytes, we used a CCR2 depleting antibody MC-21 in our therapeutic tumor setting (63). As these CCR2^+^ monocytes are required for DIVA-induced T cell responses (7), depletion was started after the second immunization and before the infiltration of monocytes into the TME (**Figure 6A**). The rather late application of the depleting mAb for only a limited time is certainly a suboptimal experimental setup to evaluate the therapeutic efficacy of combining tumor vaccination with the depletion of CCR2^+^ cells, as we only observe minute effects on survival (**Figure 6E**) and a transient delay of tumor growth (**Figures 6C, D**). While this is suggestive of the immunosuppressive capacity of the CCR2^+^ monocytes, this combined approach appears to be insufficient to completely stop tumor growth. This is most likely due to the transient depletion of CCR2^+^ cells that reappear in peripheral blood shortly after ceasing the antibody treatment (**Figure 6B**) or tumor intrinsic adaptions alleviating the need for the CCR2/CCL2 axis. Unfortunately, the administration period of MC-21 is limited to 5 days by to the induction of neutralizing antibodies in the host mice (64) leaving us unable to clarify this with this mAb. Nevertheless, it is safe to assume that CCR2^+^ monocytes recruited into the TME contribute to the immunosuppression of cytotoxic lymphocyte functions and thus promoting tumor progression *in vivo*. To explore the therapeutic potential of combined vaccination with CCR2 blockade to effectively prevent infiltration of immunosuppressive monocytes into the TME alternative CCR2- or CCL2-blocking agents are needed allowing prolonged application to achieve durable effects. Here, the selective CCR2 antagonist RS504393 that inhibits the infiltration of immunosuppressive MDSC into the TME in a bladder cancer mouse model (65) or CCL2 specific antibodies such as C1142 inhibiting tumor progression in a glioma model (66) might be interesting novel agents.

Taken together, our transcutaneous immunization method DIVA^2^ displays a promising approach to generate high quality antigen-specific T cells enabling tumor control in a therapeutic setting. Thorough analysis of the induced TME identifies immunosuppressive CCR2^+^ monocytes as important counterparts of antigen-specific T cells limiting their anti-tumor capacity. Therefore, besides boosting tumor specific cytotoxic T cell responses, future immunotherapeutic vaccination approaches must focus on the immunosuppressive TME, including CCR2^+^ monocytes.

## 4 Materials and methods

### 4.1 Mice

C57BL/6 mice - purchased from the Envigo Laboratory (Envigo, Indianapolis, USA) - were used at the age of 8-10 weeks. All animal studies were conducted according to the national guidelines and were reviewed and confirmed by an institutional review board/ethics committee headed by the local animal welfare officer (Dr. M. Fassbender) of the University Medical Center (Mainz, Germany). The responsible federal authority (Federal Investigation Office Rhineland-Palatinate, Koblenz, Germany) gave approval of the animal experiments (Approval ID: AZ 23 177-07/G18-1-096).

### 4.2 Transcutaneous immunization

Immunizations were performed under isoflurane/oxygen anesthesia (0.5% oxygen, 2.5% isoflurane). For DIVA (7), both ears of the mice were treated, each with 25 mg dithranol in vaseline (0,3 µg/mg, manufactured by the Pharmacy of the UMC Mainz according to European Pharmacopeia (Ph. Eur.) standards) corresponding to a total amount of 8 µg dithranol per ear. After 24 h the treatment with 50 mg IMI-Sol formulation (67) containing imiquimod (5% w/w, manufactured by Jonas Pielenhofer and Sophie Luise Meiser, JGU Mainz, Germany) on each ear was conducted, followed by the application of officinal cremor basalis together with OVA_257-264_ and OVA_323-337_ (100 µg each, from peptides & elephants, Henningsdorf, Germany) on each ear. For DIVA^2^, immunization was repeated after 7 days.

### 4.3 Tumor cell inoculation

For the inoculation of MC38mOVA tumor cells (68) mice were anesthetized with isoflurane and oxygen as indicated above. 5x10^4^ tumor cells were inoculated subcutaneously (*s.c.*) on the shaved right flank. After 6 days tumors were palpable and measured three times per week with a digital caliper. The survival of the mice was monitored. Tumor experiment was stopped when the tumor volume of a mouse exceeded 600 mm^3^ or when ulceration of a tumor was observed.

### 4.4 Depletion of CCR2^+^ monocytes

When indicated mice were injected intravenously (*i.v.*) with CCR2-depleting antibody (clone MC-21, 20 µg in PBS, once per day on day 15-19 after inoculation of tumor cells, provided by Matthias Mack, Regensburg, Germany).

### 4.5 Preparation of single cell suspensions from blood, tumor and spleen

To obtain peripheral blood samples tail vein incision was performed. Red blood cells were removed by a hypotonic lysis step with ACK buffer. Tumors were digested with Collagenase type 4 (800 U/ml, Worcester, Pappenheim, Germany) and DNAse type I (100 µg/ml, Sigma-Aldrich, Taufkirchen, Germany) on a gentleMACS Octo Dissociator (Miltenyi Biotec, Bergisch Gladbach, Germany). Spleens were grinded on a 70 µm cell strainer with a syringe plunger, followed by a hypotonic lysis with Gey´s lysis buffer for 2 min.

### 4.6 Flow cytometric analysis of circulating specific T cell responses

For flow cytometric analysis of DIVA induced specific T cell responses, blood cells were prepared as mentioned above and incubated for 30 min at 4°C with fluorescently labeled antibodies against CD8 (Pacific Blue-conjugated, clone 53-6.7), CD44 (APC-conjugated, clone IM7) and CD62L (FITC-conjugated, clone MEL-14). CTLs specific for H-2K^b^-OVA_257-264_ were detected by H2-K^b^ tetramer (PE-conjugated, own product). Dead cells were detected using eBioscience™ fixable viability dye (eFluor780-conjugated). Measurements were performed with a LSRII Flow Cytometer and FACSDiva software (BD Pharmingen, Hamburg, Germany).

### 4.7 Flow cytometric analysis of tumor-infiltrating leukocytes

For flow cytometric analysis of tumor-infiltrating myeloid cells, tumor single cell suspensions were incubated for 30 min at 4°C with fluorescently labeled antibodies against CD45 (BUV805-conjugated, clone 30-F11), CD3 (PE-Cy5-conjugated, clone 145-2C11/17A2), CD19 (PE-Cy5, clone 6D5), NK1.1 (PE-Cy5-conjugated, clone PK136), MHCII (BV786-conjugated, clone M5/114.15.2), CD11c (APC-R700-conjugated, clone N418), CD11b (BV605-conjugated, clone M1/70), Ly6C (BV580-conjugated, clone HK1.4), Ly6G (BV750-conjugated, clone 1A8), F4/80 (BB790-conjugated, clone T45-2342), XCR1 (BV650-conjugated, clone ZET), CD24 (BUV395-conjugated, clone M1/69), CD64 (BUV737-conjugated, clone X54-5/7.1), FcγRIε (PE-Dazzle594-conjugated, clone Mar1). Tumor-infiltrating lymphoid cells were incubated with fluorescently labeled antibodies against CD45 (BV421-conjugated, clone 30-F11), CD3 (PE-Cy5-conjugated, clone 145-2C11/17A2), CD8 (BV480-conjugated, clone 53-6.7), CD44 (BV786-conjugated, clone IM7), CD62L (FITC-conjugated, clone MEL-14), H2-K^b^-OVA_257-264_ tetramer (PE-conjugated, own product), PD1 (PE-Cy7-conjugated, clone RMP1-30), CTLA-4 (BV605-conjugated, clone UC10-4F10-11) and Lag3 (PerCP eFl710-conjugated, clone eBioC9B7W). In both panels, dead cells were detected using eBioscience™ fixable viability dye (eFluor780-conjugated). Measurements were performed with a FACSymphony Cytometer and FACSDiva software (BD Pharmingen, Hamburg, Germany).

### 4.8 IFN-γ ELISpot assay

Production of IFN-γ was assessed by IFN-γ-ELISpot assay as described previously (7). 96-Well MultiScreenHTS IP plates (0.45 mm, Merck Millipore, Darmstadt, Germany) were coated over night at 4°C with murine anti-IFN-γ antibody (clone AN18, Mabtech, Nacka Strand, Sweden). The membrane was blocked with IMDM + 10% FCS for at least 60 min at 37°C, whereupon 5x10^5^ splenocytes or *ex vivo* tumor cells were added in the absence or presence of OVA_257-264_ or OVA_323-337_ (each 1 μM). After 20h incubation at 37° C the plate was washed and stained with a biotinylated anti-IFN-γ antibody (clone R4-6A2, Mabtech, Nacka Strand, Sweden). For detection of produced IFN-γ Vectastain ABC Kit (Vector Laboratories, Burlingame, USA) together with AEC (Sigma-Aldrich, Taufkirchen, Germany) was used as described in manufacturer´s instruction. The analysis of the ELISpot plate was performed with an AID iSpot ELISpot reader (AID Autoimmun Diagnostika, Straßberg, Germany).

### 4.9 Single-cell mRNA-sequencing of tumor-infiltrating leukocytes

Tumor-infiltrating leukocytes were isolated from tumor cell suspensions by MACS sorting using CD45 MicroBeads and LS Columns (both from Miltenyi Biotec, Bergisch Gladbach, Germany). The viability analysis, single cell capturing and mRNA isolation was performed with a BD Rhapsody™ Single-Cell analysis system (BD Biosciences, Franklin Lakes, USA), following the manufacturer´s guidelines. Each sample was tagged with a unique sample tag allowing multiplexing of samples on the same single cell capturing cartridge. DNA libraries for Whole Transcriptome Analysis (WTA) and Sample Tags were created, following the BD Rhapsody System mRNA Whole Transcriptome analysis (WTA) and Sample Tag Library Preparation protocol with the BD WTA Amplification Kit (BD Biosciences, Franklin Lakes, USA). The sample preparation was performed in cooperation with the Research Center for Immunotherapy (FZI) Core Facility NGS of the Johannes Gutenberg-University Mainz. Sequencing was performed by Novogene Co. Ltd. (Cambridge, UK).

### 4.10 Bioinformatic analysis of the scRNA-seq data

Single cell RNA-seq libraries were constructed according to BD Rhapsody WTA library preparation protocol. Short read sequences were processed using the Seven Bridges analytic workflow (version 1.9). Two to three independent biological replicates of single-cell libraries were sequenced on Illumina NovaSeq6000 sequencer instruments. The dataset was annotated to gene-level information based on ENSEMBL [v92]. Quality control was performed on each dataset independently to remove poor-quality cells, using the scater package (version 1.24.0) (69). The proportion of mitochondrial gene content was used as a proxy for damaged cells, using three median absolute deviations as a threshold, following the recommendations of the OSCA resource (https://bioconductor.org/books/release/OSCA/ (70). Normalization of cell-specific biases was performed on the sets of cells passing the quality control filters using the deconvolution method of Lun et al. (version 1.24.0) (71). Counts were divided by size factors to obtain normalized expression values that were log-transformed after adding a pseudocount of one. Integration of different biological samples was performed using the MNN method (72). Highly variable genes were identified on the pooled set of cells after decomposing the per-gene variability into technical and biological components based on a fitted mean-variance trend. Next, we performed dimension reduction and clustering. Principal Component Analysis (PCA) was performed and provided as initialization to the t-SNE algorithm/UMAP algorithm (73) to obtain a reduced dimensionality representation of the data. Clustering was performed using the highly variable genes (HVGs), building a shared nearest neighbor graph (74). The Walktrap community finding algorithm was applied to determine cluster memberships. Cluster annotations were initially performed with the SingleR package (version 1.10.0) (75), using the ImmGen database as a reference. Annotations were also refined manually based on canonical markers, in conjunction with marker genes identified programmatically with the scran function “findMarkers”. Complementary exploration was performed with iSEE (version 2.8.0), which was adopted to generate most single-cell data visualizations (76). Differential state analyses, as a combination of differential expression analysis and differential abundance analysis, were conducted in the pseudobulk framework, following the implementation of the muscat package (version 1.10.0) (77).

### 4.11 Analysis and visualization of flow cytometry data

Flow cytometry data were analyzed and visualized using FlowJo (version 10.8.2, Mac OS Ventura). For dimensionality reduced visualization of tumor-infiltrating myeloid cells 1x10^4^ CD45^+^ cells were first downsampled by running the DownsampleV3 plugin. After concatenating the obtained fcs files, t-SNE plots were calculated by running the t-SNE plugin. For clustering of different myeloid populations, the FlowSOM plugin was performed and applied onto the t-SNE plots.

### 4.12 Statistical analysis

The statistical analysis was performed using GraphPad Prism (version 9.4.1 for Mac OS Ventura, GraphPad Software, San Diego California, USA). Multiple comparisons between more than two groups were performed by two-way ANOVA with Sidak’s multiple comparisons adjustment. When sample numbers in multiple comparisons were different, one-way ANOVA with Kruskal-Wallis test was performed. Comparisons of two groups were performed by unpaired Mann-Whitney test. When sample numbers of two compared groups were different, unpaired t test with Welch´s correction was performed. Comparisons of survival curves were performed by Log-rank (Mantel-Cox) test. The significance level was determined as a p value α=0,05.

## Data availability statement

The datasets presented in this study can be found in online repositories. The names of the repository/repositories and accession number(s) can be found below: Gene Expression Omnibus (GEO) under accession number GEO GSE239384.

## Author contributions

JB: Visualization, Methodology, Investigation, Writing - original draft. A-KH: Investigation, Methodology, Visualization, Writing – original draft. LS: Resources, Writing – review & editing, Investigation. DA: Methodology, Writing – review & editing. MK: Methodology, Supervision, Writing – review & editing. MS: Supervision, Writing – review & editing. FM: Methodology, Software, Supervision, Validation, Writing – review & editing. JP: Investigation, Resources, Writing – review & editing. SLM: Methodology, Resources, Writing – review & editing. PL: Funding acquisition, Methodology, Resources, Supervision, Writing – review & editing. MM: Methodology, Resources, Writing – review & editing. SM: Funding acquisition, Methodology, Resources, Writing – review & editing. HP: Conceptualization, Investigation, Methodology, Writing – review & editing. HS: Funding acquisition, Methodology, Resources, Supervision, Writing – review & editing. MR: Conceptualization, Funding acquisition, Methodology, Project administration, Resources, Supervision, Validation, Visualization, Writing – review & editing.
