# Supplementary material for: Tumor-infiltrating CCR2+ inflammatory monocytes counteract specific immunotherapy
Source: Front Immunol. 2023 Oct 2;14:1267866. doi: 10.3389/fimmu.2023.1267866 (PMC10577317; doi:10.3389/fimmu.2023.1267866)
Supplement: Supplementary file 1 [file DataSheet_1.pdf]

Supplementary Material

Tumor infiltrating CCR2$^+$ inflammatory monocytes counteract specific immunotherapy

Authors:

Joschka Bartneck, Ann-Kathrin Hartmann, Lara Stein, Danielle Arnold-Schild, Matthias Klein, Michael Stassen, Federico Marini, Jonas Pielenhofer, Sophie Luise Meiser, Peter Langguth, Matthias Mack, Sabine Muth, Hans-Christian Probst, Hansjörg Schild, Markus P. Radsak*.

*Correspondence: Radsak@uni-mainz.de

1 Supplementary Material and Methods

In vitro proliferation assay of OT-I T cells

In vitro proliferation assays with OT-I T cells were performed for analyzing the presentation of the ovalbumin epitope SIINFEKL on MHC-I molecules on the surface of MC38 and MC38mOVA cells *in vitro* and *ex vivo*. Therefore, splenocytes from OT-I mice were prepared as indicated above and labeled with 5 µM CFSE in 1x PBS for 4 min at 37 °C. After a washing step with FCS, cells were counted, resuspended in RPMI (10 % FCS, 50 µM β-Mercaptoethanol) and adjusted to a concentration of $1x10^7$ cells/ml. Tumor cells were prepared as indicated in the manuscript and adjusted to a concentration of $1x10^7$ cells/ml. Coculture of splenocytes and tumor cells was performed for 48 h at 37 °C at indicated ratios. After incubation, the cells were harvested and stained extracellularly with FACS antibodies against the epitopes CD45 (BV785-conjugated) and CD8 (BV510-conjugated) for flow cytometry analysis of proliferated T cells. Proliferation was determined by analyzing the CFSE signal in the FITC channel. Dead cells were detected using eBioscienceTM fixable viability dye (eFluor780-conjugated).

Flow cytometry analysis of stimulated CD4$^+$ and CD8$^+$ T cells

For T cell phenotyping during depletion of CCR2$^+$ cells in a tumor experiment (Figure 6), blood cells were prepared as mentioned in material and methods and incubated for 30 min at 4 °C with fluorescently labeled antibodies against CD8 (BUV805-conjugated, clone 53-5.7), H2-Kb-OVA257-264 tetramer (PE-conjugated, own product), CD44 (BV786-conjugated, clone IM7), CD62L (BV421-conjugated, clone MEL-14) and KLRG1 (BV711-conjugated, clone 2F1). For detection of regulatory T cells and intracellular cytokines the Foxp3/Transcription Factor Staining Buffer Set (eBioscience) was used with the fluorescently labeled antibody against FoxP3 (FITC-conjugated, clone FJK-16s). For detection of cytokines, cells were stimulated for 4 h at 37 °C with restimulation media containing 50 ng/ml Phorbol-12-myristat-13-acetate (PMA), 1µg/ml ionomycin and monensine solution (1:1000) and stained with intracellular antibodies against IFN-γ (BUV737-conjugated, clone XMG1.2), TNF-α (BV480-conjugated, clone MP6-XT22). Dead cells were detected using eBioscienceTM fixable viability dye (eFluor780-conjugated).

Bioinformatic analysis of the scRNA-seq data

The bioinformatic evaluation of the scRNA-seq data was performed analogously to the evaluation in the manuscript.

2 Supplementary Figures

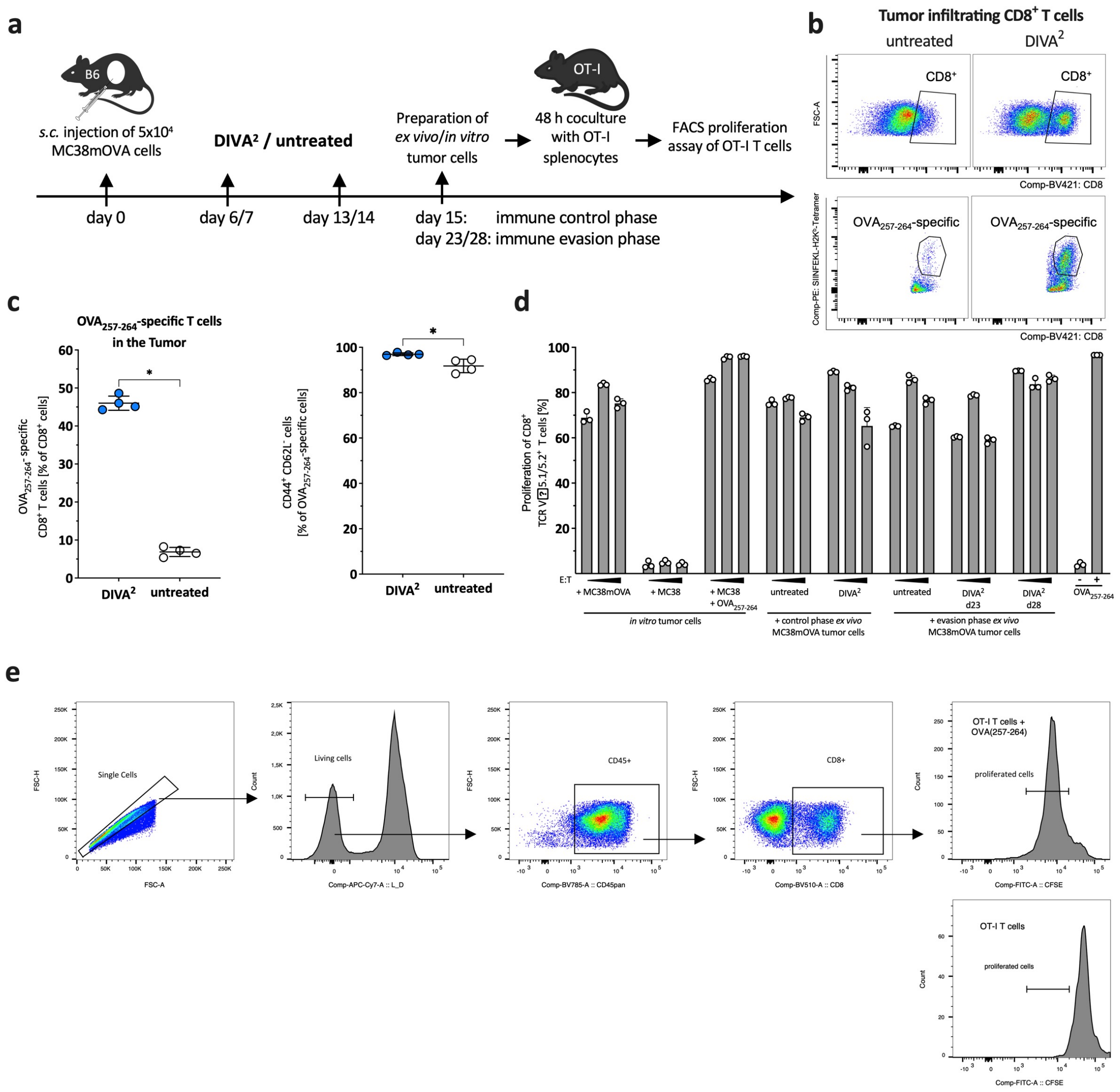

Suppl. Fig. 1: DIVA^2-induced Ova-specific T cells do not cause antigen loss on MC38mOVA tumor cells.

a) Schematic overview of the application pattern for DIVA^2 in a therapeutic tumor setting. *Ex vivo* tumor cell suspensions were prepared by MACS separation of CD45$^+$ cells at day 15 (immune control phase), day 22 or day 28 (both immune evasion phase). *Ex vivo* and *in vitro* tumor cell suspensions were co-cultured for 48 h with CFSE-labeled OT-I splenocytes. T cell proliferation was assessed by flow cytometry. b) Representative flow cytometry dot plots of tumor-infiltrating CD8$^+$ and OVA$_{257-264}$-specific CD8$^+$ T cells of untreated and DIVA^2 treated mice on day 23. c) OVA$_{257-264}$ -specific CD8$^+$ T cells and their activation state were determined by flow cytometry on day 23. Depicted are the individual values with mean and SD. Statistical analysis using Man Whitney test, n=4 d) *In vitro* proliferation assay of CFSE-labeled OT-I splenocytes. Proliferation of TCRVβ5.1/5.2$^+$ T cells was determined after 48 h Co-culture by flow cytometry. Effector:Target (E:T)-ratios of OT-I splenocytes to tumor cell suspension were 4,5:1; 13,5:1; 40,5:1. Peptide Control samples were cultured with OVA$_{257-264}$ peptide (SIINFEKL, 10 ng/ml). Shown are individual values of separate *in vitro* co-cultures, mean and SD, n=3. e) After gating on single cells and living cells, CD45+ immune cells and CD8+ T cells were discriminated. The proliferation of OT-I T cells was determined by analysis of the CFSE signal in the FITC channel.

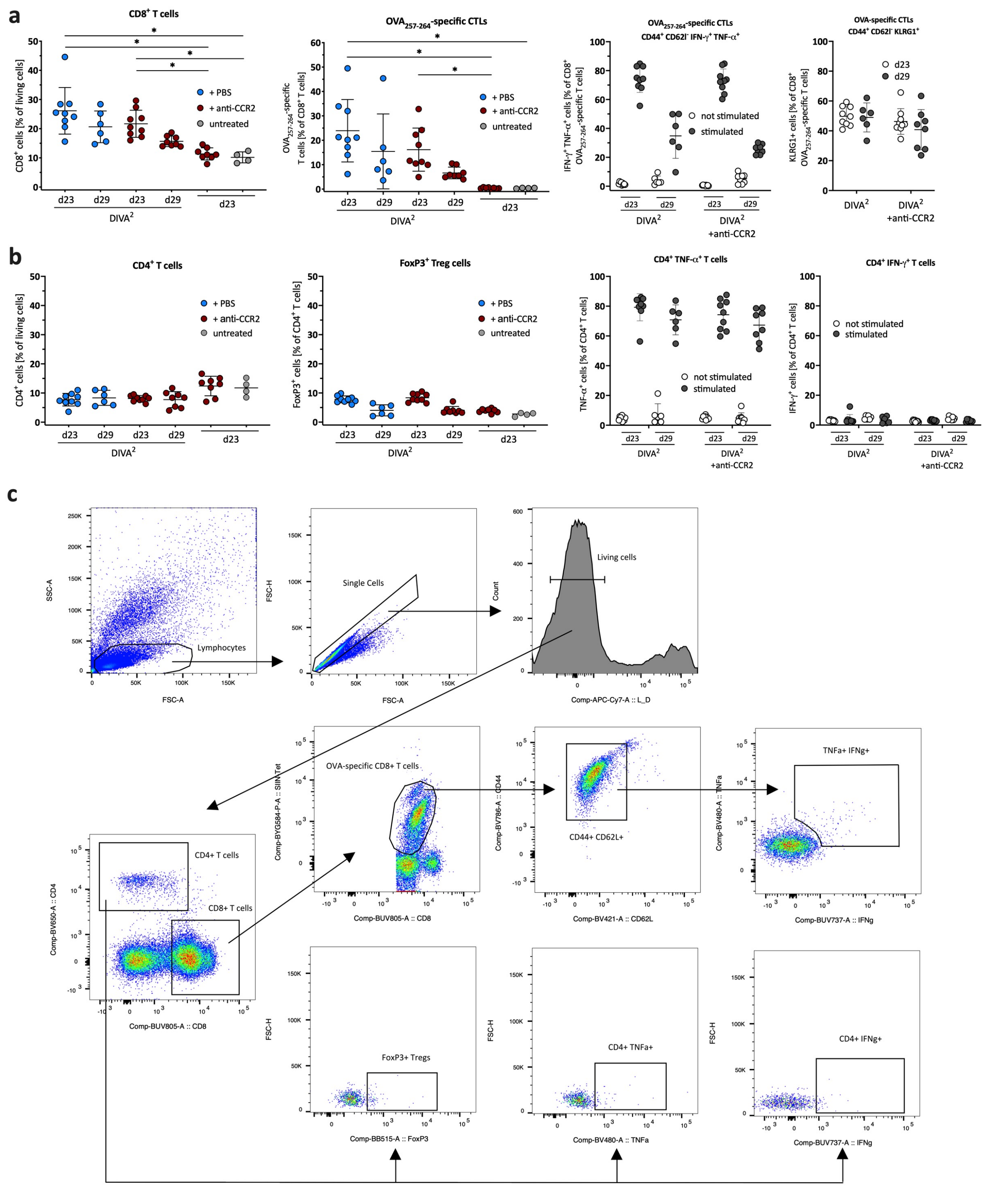

Suppl. Fig. 2: Systemic depletion of CCR2+ cells by MC-21 does not lead to depletion of T cells.

T cell phenotyping of $CD8^+$ T cells in a) and $CD4^+$ T cells in b) was determined by flow cytometry at day 23 and 29. Stimulation of $CD4^+$ and $CD8^+$ T cells for assessing cytokine production by flow cytometry was performed for 4 h at 37 °C and stimulation with 0,5 µg/ml Ionomycin, 50 ng/ml PMA and 2 µM Monensin. c) Cells were gated on lymphocytes, single cells and living cells. After discrimination of $CD8^+$ and $CD4^+$ T cells, the $CD8^+$ T cells were gated on OVA-specific T cells using a $H2K^b$-$OVA_{257\text{-}264}$ tetramer. Activated T cells ($CD44^+$ $CD62L^-$) were analyzed for IFN-γ and TNF-α production. $CD4^+$ T cells were analyzed for FoxP3 expression and production of TNF-α and IFN-γ.

a Gating strategy for Figure 2

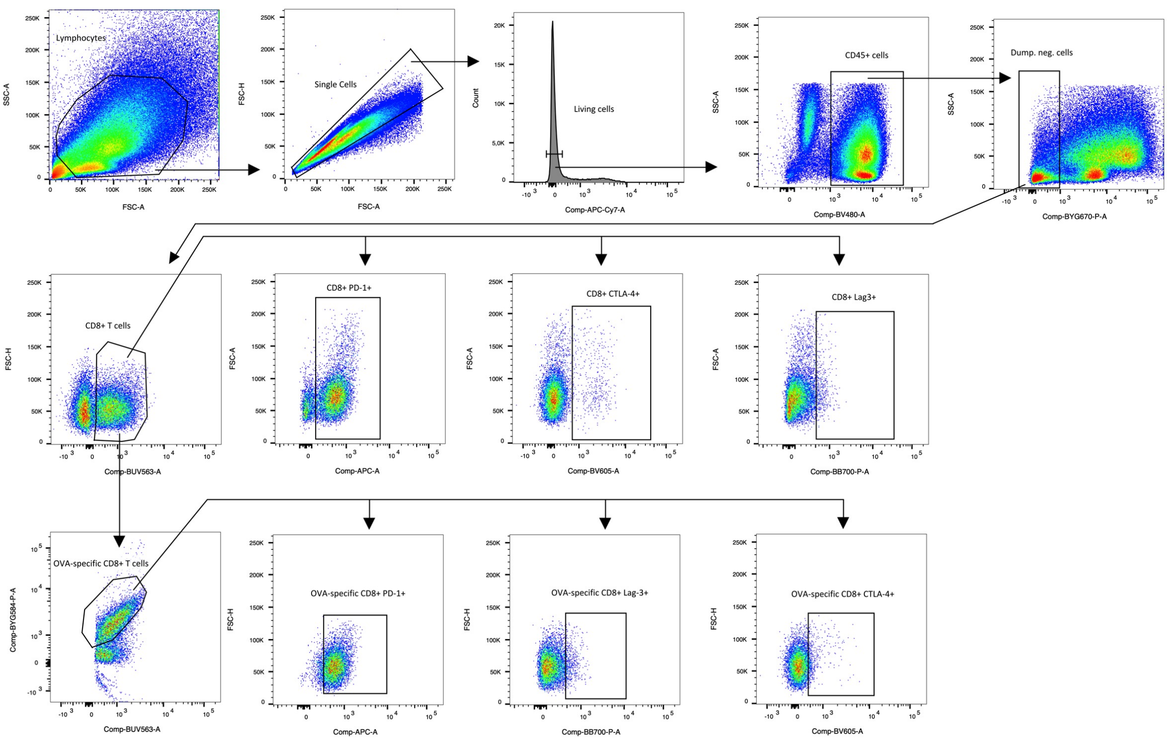

b Gating strategy for Figure 6

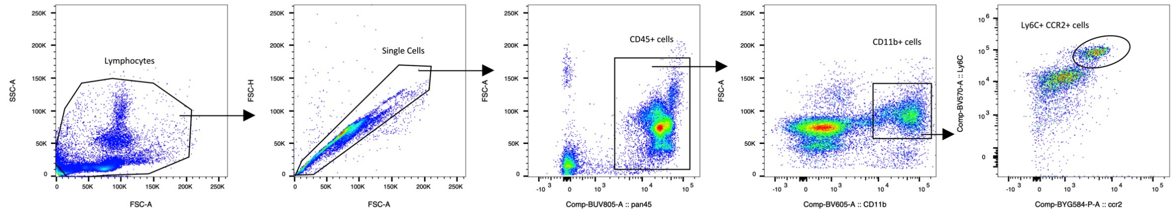

Suppl. Figure 3: Flow cytometric gating strategies for figures 2 and 6.

a) Cells were gated on lymphocytes, single cells and living cells. After gating on CD45^{+} cells and discriminating lineage-negative cells, CD8^{+} and OVA-specific CD8^{+} T cells were analyzed regarding their expression of exhaustion marker PD-1, CTLA-4 and Lag3. b) Cells were gated on lymphocytes and single cells. After gating on CD45^{+} cells, CD11b^{+} cells were further analyzed for their expression of Ly6C and CCR2 to quantify monocytes.

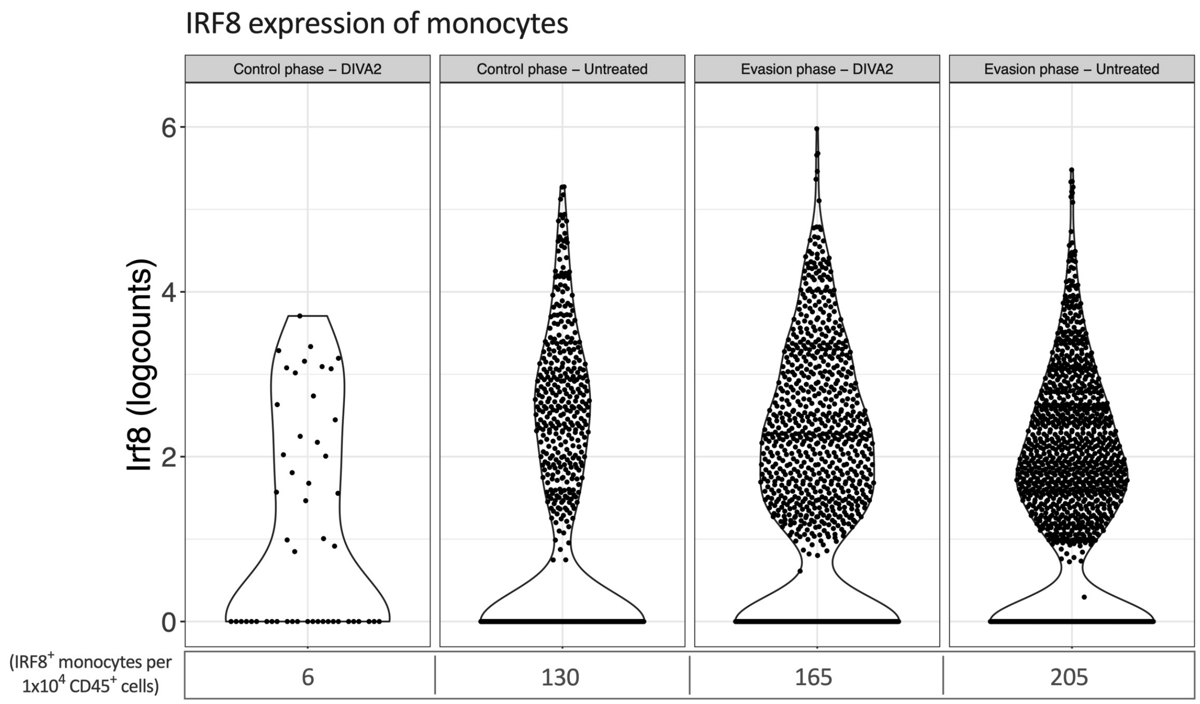

Suppl. Figure 4: Monocytes infiltrating the TME of DIVA^2-treated mice express IRF8.

ScRNA-seq-based violin plots showing the IRF8 expression of monocytes (logcounts). The cell count of IRF8 expressing monocytes is given normalized to $1x10^4$ $CD45^+$ cells in the TME. Depicted cells are accumulated per condition from n=2-3 mice.
